# Small Ruminant Models for Articular Cartilage Regeneration by Scaffold-Based Tissue Engineering

**DOI:** 10.1155/2021/5590479

**Published:** 2021-12-06

**Authors:** Liqing Peng, Bin Zhang, Xujiang Luo, Bo Huang, Jian Zhou, Shuangpeng Jiang, Weimin Guo, Guangzhao Tian, Zhuang Tian, Shi Shen, Yangyang Li, Xiang Sui, Shuyun Liu, Quanyi Guo, Haibo Li

**Affiliations:** ^1^Department of Orthopedics, First People's Hospital of Shuangliu District, No. 120, Chengbeishang Street, Shuangliu District, Chengdu 610200, China; ^2^Department of Orthopedics, The First Medical Centre, Chinese PLA General Hospital, Beijing Key Lab of Regenerative Medicine in Orthopedics, Key Laboratory of Musculoskeletal Trauma & War Injuries PLA, No. 28 Fuxing Road, Haidian District, Beijing 100853, China; ^3^Center for Biomedical Materials and Tissue Engineering, Academy for Advanced Interdisciplinary Studies, Peking University, No. 205 Chengfu Road, Haidian District, Beijing 100871, China

## Abstract

Animal models play an important role in preclinical studies, especially in tissue engineering scaffolds for cartilage repair, which require large animal models to verify the safety and effectiveness for clinical use. The small ruminant models are most widely used in this field than other large animals because they are cost-effective, easy to raise, not to mention the fact that the aforementioned animal presents similar anatomical features to that of humans. This review discusses the experimental study of tissue engineering scaffolds for knee articular cartilage regeneration in small ruminant models. Firstly, the selection of these scaffold materials and the preparation process *in vitro* that have been already used *in vivo* are briefly reviewed. Moreover, the major factors influencing the rational design and the implementation as well as advantages and limitations of small ruminants are also demonstrated. As regards methodology, this paper applies principles and methods followed by most researchers in the process of experimental design and operation of this kind. By summarizing and comparing different therapeutic concepts, this paper offers suggestions aiming to increase the effectiveness of preclinical research using small ruminant models and improve the process of developing corresponding therapies.

## 1. Introduction

Local damage to articular cartilage is caused by trauma, strain, and degeneration. Due to the lack of blood vessels and neurotrophic function of articular cartilage and the poor ability to self-heal [1], the lesion often progresses gradually. The development of rough and fibrotic cartilage surface structures, sclerotic subchondral bone cysts, and surrounding osteophytes and intra-articular inflammation can all cause clinical symptoms and reduce quality of life [2]. Therefore, reparative interventions for cartilage injuries or defects in the initial stage can control or delay further development of the lesion and reduce the requirements for knee arthroplasty [3]. To achieve this goal, a large number of studies have been conducted on cartilage repair using tissue engineering methods. As an environment for cell growth, proliferation, and differentiation, scaffolds have always been an important research object in this field, and many methods and biomaterials have been developed to prepare scaffolds with confirmed chondrogenic effects. Although many scaffolds have achieved ideal results *in vitro*, there is still much work to be done in clinical translation. To achieve effectiveness and safety before clinical use in humans, animal studies are essentially required to evaluate new therapies in cartilage lesions in weight-bearing areas [4].

There are no perfect animal models for the preclinical research. Even though nonhuman primates maybe the most similar models to that of humans, ethical issues hinder them from a convenient animal model [5]. Therefore, small animals such as rodents, rabbit, and guinea pigs become the most widely used models for investigation of cartilage repair strategies. However, their cartilage thickness and load characteristics made it difficult for them to be a translational model for cartilage surface repair. Secondly, some small animals such as rodent can maintain unclosed growth plates in their cells from the metaphysis that may provide regenerated cartilage and correspondingly change research results [6]. Different from small animals, large animal models include dogs, small ruminants (goats and sheep), and horses can provide physiological microenvironment closer to those of human cartilage and a similar biomechanical environment in load and friction due to their body shape, gait, and movement characteristics. Furthermore, larger body size facilitates researchers to create cartilage defects, and a thicker cartilage layer can minimize the invasion of subchondral bone plate in full-thickness cartilage defect researches. With each advantage and disadvantage of large animals, we found that goats and sheep have been the most widely used large animal models in the past 13-17 years [7–9]. But in the study of use small ruminants' models, the researchers employed experimental designs and surgical operations in various ways; this review elaborates on the scaffolds, surgical process, postoperative management, and evaluation of experimental indicators in order to provide researchers who need to conduct relevant animal experiments a reference in terms of designs and methods.

## 2. Methods

We performed a search (up to September 2021) in the PubMed and Medline to identify available scientific articles about the experiments of cartilage repair by tissue engineering scaffold in sheep or goat models. For the purpose of this review, several combinations of the following keywords have been used: sheep, goat, ovine, caprine, large animal, scaffold, knee, osteochondral, chondral, subchondral, resurfacing, repair, defect, cartilage, and tissue engineering; only *in vivo* model studies were finally considered. Also, we tabulated the information pertaining to the age and species of animals utilized, group number, defect dimensions, location, and study duration (Table 1). The emphasis of this review is on knee joint cartilage defect regeneration, therefore chondral or osteochondral defects created in humeral head or femoral head are not include in the review. Also, experiments for meniscal regeneration involving articular cartilage are excluded.

### 2.1. *In Vitro* Scaffold Preparation

This review introduces scaffold experiments performed in sheep and goats with different materials and structures, with or without cells and other components in repair of chondral and osteochondral tissues. Most scaffolds are made of natural materials because of their relatively high biocompatibility, and products of degradation have less influence on the microenvironment of the repair area. With the development of materials and technology, there are more and more scaffolds made of synthetic materials that have stronger mechanical properties and are more operable in the preparation progress.

**Figure 1:**
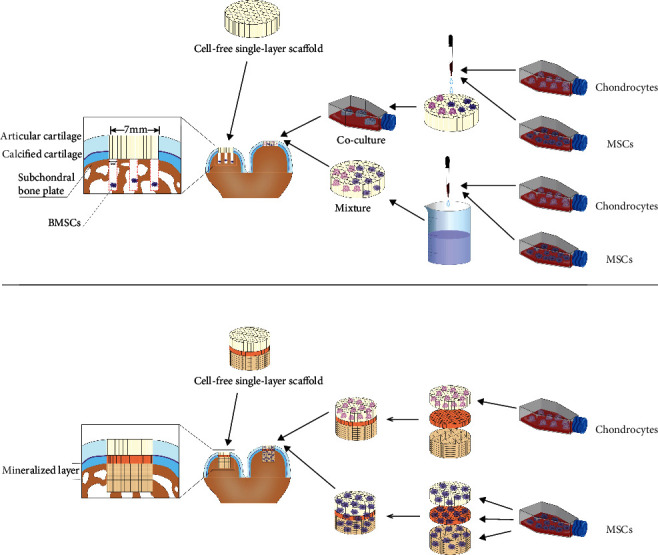
Schematic diagram of full-thickness cartilage or osteochondral scaffold loading of cells *in vitro* and implantation in the defect area.

Single-layer scaffolds for repairing cartilage are mostly suitable for partial- or full-thickness cartilage damage that has not yet involved the subchondral bone (Figure 1). Most of these scaffolds are prepared with a single ingredient; natural materials, such as collagen, gelatin, hyaluronic acid (HYA), and hydrogel, are mostly used to repair such cartilage defects. Besides, de Barros et al. chose a thrombin-like compound derived from snake venom as a scaffold to repair sheep cartilage [10]. Beck et al. reported the use of a collagen scaffold combined with an autologous matrix-induced chondrogenesis (AMIC) technique to verify reparative effect [11]. Several scaffolds prepared from single-component synthetic materials have been used to demonstrate cartilage defect repair, including nonwoven filamentous polyethylene terephthalate [12] and pure polyglycolic acid (PGA) [13, 14]. Because the microenvironment after implantation is similar to natural cartilage that lacks vascular and neurotrophic factors, it is quite essential for the scaffold material to maintain a higher capacity for cell recruitment and chondrogenesis. Meanwhile, scaffolds that are biomimetic in both structure and composition facilitate cell adhesion. Many researchers use extracellular matrix (ECM) as a scaffold material as well because it plays a crucial role in maintaining stem cell characteristics and regulating stem cell activation. Depending on the composition and properties of the ECM in hand, various differentiation pathways can be triggered. In addition to the physical characteristics of the ECM, growth factors and glycoproteins presented in the ECM act as significant regulators to balance the activation and quiescence of stem cells [15].

In the later stages of cartilage damage and osteoarthritis, when the damage is often accompanied by local subchondral bone fibrosis, bone nodule formation, and cystic changes, simply repairing the cartilage layer may not be able to achieve the purpose of treatment. In this case, the use of an osteochondral scaffold becomes a rational repair method. Due to different anatomy, regeneration microenvironment and mechanical requirements of cartilage and bone and multilayer scaffolds prepared with different components are mostly used for repair. Two biphasic scaffolds, “Chondromimetic™” and “TruFit™,” were compared in the repair of goat knee cartilage [16]. Kon et al. biologically modified a coral scaffold (with and without HYA impregnation) to prepare biphasic scaffolds and drilled channels in the cartilage or bone phase to increase porosity. Such design may reduce the content of scaffold material to facilitate the repair process and therefore improve the biomechanical microenvironment of the surrounding cartilage [17]. Filardo et al. constructed biphasic scaffolds with different components: the bony layer consists of a composite made of 1.25% alginate and 4% hydroxyapatite (HA), while the chondral layer consists of 1% alginate and 0.5% HYA [18]. Biphasic scaffolds were also used by Cunniffe Gráinne, who repaired cartilage and bone with porous scaffolds composed of growth plate ECM which was later overlaid with articular cartilage ECM [19]. Vikingsson et al. prepared a polycaprolactone (PCL) scaffold attached to a poly (L-lactic acid) (PLLA) pin to reduce subchondral nodule formation [20]. Levingstone et al. prepared multilayered scaffolds consisting of a bone layer (type I collagen with HA), intermediate layer (type I collagen and HYA), and a cartilage layer (type I and type II collagen). Histological analysis showed successful restoration of the anatomy regarding tidemark of the calcified cartilage layer [21]. Getgood et al. added RhFGF18 or BMP-7 to the scaffolds made of “Chondromimetic™.” After treatment with scaffolds loaded with rhFGF18, staining for collagen VI and collagen II was positive around cells in the repaired tissue, while staining for collagen I was decreased [22]. TGF-*β* 3 and IGF-1 were added dropwise after scaffolds were prepared with chondrogenic microspheres and osteogenic microspheres, and these scaffolds were compared with the microfracture technique [23]. The bone layer of the existing osteochondral scaffold is often used alone or in combination with natural materials to enhance hardness and provide a microenvironment for osteogenesis. Even if the same material is used in the bone and cartilage layers of the scaffolds, the proportion will still be adjusted during preparation in order to approximate the mechanical properties of the tissue in situ. However, further research is still required to exclude any potential long-term toxicity that may be caused by the scaffold given that the biodegradation rates and products of various components are different. Small ruminants have a relatively long lifespan (10-12 years) [24], so these models can provide sufficient time to observe whether the scaffolds can maintain ideal morphology and provide a satisfying growth environment for cells in the process of cartilage regeneration.

In addition to differences in materials and structures, the biggest difference between various tissue engineering scaffolds lands on the issue of whether to load cells on the scaffolds. At present, most cell-loaded scaffolds are prepared using materials that are similar to the natural cartilage matrix in terms of water absorption capability, porosity, and biocompatibility, providing cells with a good space for internal adhesion, growth, and biological activity as well as chondrogenic capability. Autologous chondrocyte implantation has a good reparative effect in cartilage defects [25]; hence, many cartilage defects were repaired by a complex of autologous chondrocytes and scaffolds after coculturing in the early stage of this field. Yet, some researchers chose to load with other components: Kon et al. loaded PRP in three-layer scaffolds prepared by collagen I and HA [26]; Getgood et al. combined PRP with a biphasic collagen/GAG scaffold [27]; Jubel et al. repaired a knee cartilage defect in sheep with chondrocyte-derived scaffold-like implants [28]. Furthermore, in later studies in the field, the utilization rate of mesenchymal stem cells (MSCs) gradually increased because they come from a wide range of sources, such as bone marrow and adipose tissue. It is crucial to note that stem cells have multiple differentiation potentials [29, 30]; they can differentiate into various types of cells and form functional and morphological tissues as induced by the scaffold materials or the local microenvironment, which possessing a strong ability to expand and promote cartilage and bone formation [31–33]. As a result, the MSCs can be loaded on different layers of scaffolds, making it convenient to construct the cell-scaffold complexes (Figure 1). In this stage, cells were isolated from autologous tissue. Small ruminants can provide relatively sufficient tissue and blood volumes compared with those of small animals; nonetheless, there are fewer complications at the donor site, and the cultured cells need to be passaged *in vitro* for a certain period of time (Supplementary Table (available here)). There can be a long period between cell isolation and scaffold implantation, and animals need to undergo two invasive operations for tissue harvesting and scaffold implantation. If an ideal repair can be achieved through a single operation, the corresponding approach will present certain advantages in terms of time and cost after translation to the clinic.

### 2.2. *In Vivo* Study Performed in Small Ruminant Models

#### 2.2.1. Physiological Characteristics of Small Ruminants

Body size and weight are important criteria for selecting animal models for orthopedic research [34]. Compared with dogs and smaller animals, knee joints of small ruminants display strong anatomic similarity to humans, and a thick cartilage makes it easier to create a partial- or full-thickness defect, which facilitate their use as translational models [35]. The bone mineral density of the mineralized layer in mature sheep is similar to that of the medial femoral condyle in humans (1.17 ± 0.15 g/cm^3^) [36]; such similarity may help the removal of cartilage thoroughly and avoid involving the subchondral bone plate. Due to regional and subspecies differences, body weight and local anatomical structure vary among mature small ruminants. For example, the femoral condylar cartilage thickness in mature sheep has been reported to be 1.68 mm [37], 0.4-1 mm [38], and 1.2 mm [39]. In goats, thickness of cartilage is 0.8-2.0 mm [40] and 1.1-1.4 mm [41]. Limited data have shown that animals used in European and North American countries are relatively heavier than other continents, ranging from 40 kg to 74.54 ± 16.55 kg, while those used in South America and Asia are relatively lighter, ranging from 20.2 ± 5.3 kg to 58.02 ± 11.38 kg. Differences in body weight may result in different volumes of local anatomical structures; however, we found that light models can also achieve a large range of defects in the load-bearing area of the femoral condyle (Table 1). Skeletal maturity in sheep and goats typically occurs between 24 and 36 months [36]. When it comes to the age of animals, defects in the skeletally mature animals were reported to be “unsuccessfully repaired” than in immature animals [42]; thus, most researchers tend to choose mall ruminants over 2-3 years of age not only because the repair potential of cartilage caused by differences in cartilage matrix, cells, and lipid content is age-related but also mature small ruminants have a similar metabolic and bone remodeling rates to that of adult humans [43].

#### 2.2.2. Group Design

When determining the number of animals in each group, the 3Rs principle (Replacement, Reduction, and Refinement) should be followed as much as possible. Obviously, the greater the number of animals is, the smaller the representative errors of the statistical estimate are, and the risk of sample loss due to animal death can be avoided to a greater extent. However, because too many animals unavoidably result in ethical issues, insufficient costs, increased workload of scientific researchers, and increased difficulty in animal management, it is essential to minimize the number of animals while maintaining data sensitivity as much as possible. In each group, the aforesaid number is determined not only according to individual animal itself but also the number of defects. Under normal circumstances, the number of animals allocated to each experimental group is determined by a specific statistical test based on the strength of difference between expected results of each group; it is also recommended to carry out an appropriate experimental design with the cooperation of a statistician to produce statistically significant results [44]. In some experiments, the number in the control group is less than that in the experimental group; this is probably because an empty critical-sized chondral defect cannot heal spontaneously [45], and the results are more representative. In order to improve sensitivity of the experiment, Bate and Karp suggested that if pairwise comparisons are made, it may be a better choice to distribute the animals equally to each experimental group. If there are *t* variables, the number of animals in the control group should be $\sqrt{t}$ times that of the experimental group [46].

In animal studies, rigorous research designs should also include treatment-related repair endpoints, and different evaluation indicators need to be set at different endpoints [47]. Although scaffolds with biomimetic structures and functions have been prepared by various materials, the synergistic mechanism in the long-term process of cartilage repairing is still unclear. There are certain studies designed to observe short-term inflammation and scaffold degradation, and others designed as short-term experiments. For example, Levingstone et al. collected samples at 2 weeks to observe scaffold fixation and the early response *in vivo* [21]. Lydon et al. set a time point of 1 and 2 weeks for continuous observation of the endochondral ossification regarding osteochondral defects [48]. In three months or less, the repair tissues of large animal models are immature, including various fibrous tissues and fibrous cartilage [49]; thus, Cook et al. suggest that the study duration should be longer than 6 months in order to observe the repair effect of the scaffolds [50]. In large animal models including small ruminants, a time point of 12 months or longer designed to simulate controlled weight bearing and rehabilitation may be more valuable for clinical translation [51]. At present, most long-term evaluations end at 12 months, and the longest ended at 24 months (Table 1). Given the lifespan of small ruminants, it is quite difficult to set longer time points. But young patients undergoing cartilage repair still face the possibility of reinjury or cartilage defect recurrence in reality; consequently, assessment of the reparability of regenerated cartilage tissues is another problem that needs to be addressed in the future.

#### 2.2.3. Defect Location and Range

Clinical investigation shows that the weight-bearing areas of femoral condyle and trochlear groove are the most common areas of cartilage defects in knee joint [52, 53]; as a result, defects in animal experiments are usually placed at this site (Table 1). Femoral condyle can achieve full weight-bearing when standing and moving while the weight-bearing ability of the trochlea is intermittent, which is also a factor to be considered in the experimental design. So, the articular cartilage repair may be different depends on the topographic location [54]. As mentioned above, reports have shown that there are different cartilage thicknesses in small ruminant animals; the trochlear groove of the femur as well as the medial and lateral condyles is not flat, and the thickness of cartilage and subchondral bone varies from site to site across the surface. As to full-thickness cartilage defect, it ends at the junction of the calcified cartilage layer and the subchondral bone layer; furthermore, it should be noted that the subchondral bone structure cannot be involved [55, 56] (Figure 1). If the defect is too deep and breaks through the subchondral bone plate, causing bleeding at the bottom, this will interfere with the effectiveness of cartilage repair. To fully explore the reparative effects of the scaffolds, the defect diameter of at least 7 mm should be used. Although critical-sized defects will not heal spontaneously, with blood exudation, the result is similar to that of microfracture, and the defects in question may be filled with fibrocartilage tissue. When using cell-free cartilage scaffolds, many researchers prefer to combine them with bone marrow stimulation techniques such as microfracture or AMIC for the purpose of transferring undifferentiated pluripotent stem cells from the bone marrow cavity to the defect area for cartilage repair [57]. Combining exuded bone marrow blood with scaffolds through subchondral bone plate drilling is one way to recruit stem cells through a surgical technique. For subchondral drilling, small holes (diameter 1 mm) can significantly improve the repair of full-thickness articular cartilage defects and the recovery of subchondral bone microstructures in the sheep knee joint compared with holes larger in diameter [58, 59].

In regard to osteochondral repair, there are differences in the thickness of the calcified cartilage layer and bone plate: the mineralized layer was 727 ± 270 for sheep, and the repair depth of the osteochondral defect should be at least this deep after removal of the cartilage. Creating a defect model, especially an osteochondral defect model, involves subchondral bone tissue which is very dense and hard, so it is hard to obtain identical defects. There are no standard effective instruments; these defects are usually made using tools such as skin, corneal trephines, or drills. Schlichting et al. [60] used the SDS osteochondral transfer system (Zimmer Germany GmbH, Freiburg, Germany), which has been used clinically, and Heckelsmiller et al. developed a cartilage impact gun with a diameter of 6 mm to create cartilage defects [61]. Nonetheless, controlling the depth of the created defects relies mostly on the experience of the operator, so the uniformity and efficiency of this process are relatively low. After scaffold implantation, it is often necessary to ensure that the scaffolds will not detach in situ. Because the depth of the defect in cartilage repair studies is shallow, the risk of the scaffold becoming detached after animal activity is worthy of attention. For cartilage defects, fibrin glue is often used to cover the repair area after scaffold implantation to achieve fixation. Erggelet et al. used transosseous technology [62] in studies and anchored the scaffold through holes in the femoral bone. Some researchers have directly sutured the scaffold to the cartilage around the defect area. Zorzi et al. applied repeated flexion and extension of the sheep knee joint to check the stability of the implant after fixation [63]. To observe whether the scaffold is retained in the defect, Bornes et al. performed a second procedure one week after the first operation [64]. In osteochondral defects, the stability of the implanted scaffold is ensured by the fact that the defect is a deep cylindrical hole, and the press-fit method is usually used to fix the scaffold. In addition, the reconstructed soft tissue anatomy of the knee joint may also change the patellar trajectory on the trochlea due to scar accretion after opening the articular capsule from the side of the patella and reducing the dislocated patella. It is also necessary to select a medial or lateral patellar approach to reduce the influence of the patellar trajectory on the trochlear defect area. Perhaps a small number of animal preexperiments with budget and ethics permitting can help in making the decision. In short, when designing an experiment, understanding the physiological structure of the selected experimental animal is very helpful, such as the thickness of the cartilage in the defect area, and choose appropriate surgical instruments and techniques accordingly.

#### 2.2.4. Postoperative Management

Normally, small ruminants can stand and move on their own approximately one hour after general anesthesia. However, owing to the fact that the knee joint of sheep and goats is relatively high and close to the trunk and it is difficult to apply external fixation, the operated joints are barely immobilized and therefore allows full weight-bearing of the animals. Also, the stability of the scaffold fixation method can be used to decide whether to keep the animal in stocking—a well-acknowledged way of realizing immobilization. Pain controlling should be performed well after the operation, and appropriate analgesic schemes should be formulated. Many researchers have chosen to use nonsteroidal anti-inflammatory drugs (NSAIDs) and transdermal fentanyl patches or carprofen [65] as postoperative analgesics. Sometimes, according to specific conditions, prophylactic and postsurgical antibiotics are used from 3 days to 2 weeks. Amoxicillin and penicillin are the most commonly used antibiotics, and NSAIDs are the most frequently used postoperative analgesics because of their effective pain control and convenient administration. Antibiotics are sometimes needed to prevent infection according to the environmental conditions; however, to prevent the development of drug-resistant bacteria, it is more effective to improve the aseptic level of the operating room, feeding environment of animals, and the strict aseptic surgical technique of the operators than to use antibiotics. If needed, basic broad-spectrum antibiotics are recommended. Additionally, quinolones should be avoided because they may affect cartilage metabolism [66]. Many researchers allow animals to bear full weight or even move freely after surgery. Just like other implants, it takes time for the scaffold to integrate with surrounding tissues *in vivo*, so if the stability of the scaffold is not completely certain, it is recommended to keep the animals in captivity for a period of time and then allow them to move freely.

#### 2.2.5. Outcome Assessments

Macroscopy is the first and most intuitive examination method. The Goebel macroscopic system [67] is scored based on the color of the cartilage, number of blood vessels, smoothness of the surface, filling of the defect, and amount of adjacent cartilage. Fortier modified scoring system [68] and a scoring system proposed by Niederauer et al. [69] have been used to evaluate the gross morphology. The International Cartilage Repair Society (ICRS) scoring [70, 71] can also be applied and is currently used by most researchers to assess cartilage regeneration in defects. As to the osteochondral unit, it is formed by three structural layers (articular cartilage, calcified cartilage, and subchondral bone) [72], each of which contains different components. There are differences in the morphology and function of chondrocytes on both sides of the tidemark [73], which is a mineralized deposit that divides the interface between the articular cartilage and calcified cartilage. Histological stained can be used to assess the overall tissue, the distribution and morphology of cells in different layers, presence of abnormal calcification, and bone-cartilage interface. Furthermore, due to different contents of type I and type II collagen in hyaline cartilage, fibrocartilage, and fibrous tissue [74], the degree of hyaline cartilage formation can be determined by immunohistochemical staining for the two kinds of collagen. Some researchers [18, 75] also used osteocalcin immunohistochemical staining and vascular endothelial growth factor staining to further verify the results of bone layer repair achieved using osteochondral scaffolds. In terms of histological scoring, researchers mostly use the O'Driscoll [76], ICRS II scoring systems [77], or Pineda scoring system [78] to evaluate repaired tissues. Various scoring systems have been used to conduct semiquantitative analysis in different scoring ranges; however, subjective judgment among observers has a great influence, and only a few scoring systems have been validated, so the uniformity of the results needs to be improved [79]. Researchers may use multiple scoring systems when necessary and more than three observers with a blinding method to obtain results.

Minimally invasive and noninvasive techniques for *in vivo* measurement including CT, MRI, and arthroscopy are becoming increasingly vital in evaluating animal experiments; they can achieve the collection of results without sacrificing animals, and they are also important clinical testing methods. Moreover, both goat and sheep are feasible to the tests above, and second look arthroscopy can be performed to review integration. Micro-CT can be used to scan the distal femur and examine the thickness of the subchondral bone, the diameter of the bone trabeculae, the trabecular number, and the trabecular separation, which can present variations and remodeling of the subchondral bone during cartilage repair. Lesions in the soft tissue, cartilage, subchondral bone, and ligaments of the knee joint can be observed by MRI. Quantitative analysis of T1r and T2a MRI parameters can be performed to evaluate the effects of components on new tissue growth [80]. T2 mapping combined with delayed gadolinium-enhanced MRI of cartilage is an excellent way to discriminate between a collagen network with zonal organization and healthy cartilage [81]. Biomechanical factors can be used to evaluate the function of repaired tissue. At present, the compressive mechanical properties of repaired tissue are mainly tested, and these data are used to draw stress-strain curves and calculate the Young modulus of the osteochondral repair tissue according to the applied stress, the initial height of the specimen, the cross-sectional area of the specimen, and the compressive displacement at the point of equilibrium in the relaxation phase [82]. Alternatively, the three-stage stress-relaxation indentation scheme proposed by Stok et al. can be used to calculate the instantaneous and equilibrium compressive modulus of cartilage as well as the typical peak stress and relaxation time [83]. However, it should be noted that the elastic modulus of repaired cartilage still varies greatly in different experiments. Besides compressive mechanical, the friction, abradability, and durability of new tissues and cartilage, as an important biomechanical component of the motor system, still need further exploration. Nondestructive methods such as Gait analysis [84] and electromyography (EMG) [85] can be applied as supplementary techniques to evaluate the function of repaired tissue; they can be widely used in clinical practice when utilized appropriately.

## 3. Conclusion

With the rapid development of tissue engineering for cartilage repair, an increasing number of scaffolds have been proven to be safe and effective *in vitro* and in small animal experiments, and more large-animal experiments will be carried out. As the most widely used preclinical research model in this field, small ruminants also reveal some disadvantages. The long trochlear groove with medial and lateral ridges and the intercondylar notch width are different from human anatomy, which may cause differences in the mechanical environment. Some customized device such as postoperative joint brace for immobilization or restriction, magnetic resonance coil for MRI test may be needed, therefore unavoidably increasing the experiment budget. In animal experiments, it is difficult to collect and quantify indicators of long-term functional characteristics, such as pain and local subjective sensation. The inadequate commercial products such as detection agents and antibodies as well as gene expression microarrays may increase the limitations of immunology and molecular biology testing.

Despite these limitations, small ruminant models have been proved useful in preclinical and translational studies for articular cartilage regeneration with tissue engineering scaffold (Table 2). These models are easier to obtain ethical permission for the usage compared to dogs and horses [86]. Considering their habits and diets, small ruminants are cost-effective, relatively placid, and stifle surgery tolerance-high and are easily housed and maintained during perioperative than other large animals [87–89]. The longer lifespan can make the setting of the observation endpoint more flexible, and the larger body size can provide more tissues to isolate cells than small animals. The gross anatomy and biomechanical environment of the up-right stifle joints especially in goat models are similar to that of humans [90], thus facilitating in creating the ideal defects with enough range and depth, conducting routine diagnostic imaging, and introducing arthroscopic interventions. These characteristics of small ruminants can be used to develop the clinical diagnosis and treatment techniques, making them the ideal translational models.

Indeed, there are limitations to this review, as we failed to discuss the characteristics of sheep and goats separately and the effects of gender factors on the experiment. As the local anatomical structure of small ruminants varies among subspecies, it is necessary to establish standardized principles and procedures for establishing defect models according to the selected subspecies and to adopt reasonable surgical plans and implementation methods to ensure maximum consistency among models. Small ruminants provide a suitable model for testing tissue engineering products in preclinical and translational studies of scaffold-based methods for osteochondral defect repair. Making full use of animal models reasonably and effectively can help researchers further understand the reparability of cartilage using tissue engineering scaffolds and translate new scaffolds into clinical practice.

## Figures and Tables

**Table 1:** Animal selection and experimental implementation.

Reference	Animal	Age and weight	Group size	Location	Range	Endpoint
Beck et al. [11]	Female sheep	5-7 years, 71.2 ± 10.6 kg	36: 3 experimental groups of 12 sheep each	Medial femoral condyle	Full-thickness Diameter: 8 mm	13 and 26 weeks
Seedhom et al. [12]	Female sheep	2 years, 50-85 kg	13: 3 months, 7; 6 months, 6	Medial femoral condyle	Diameter: 7 mm	8 weeks 3 and 6 months
Erggelet et al. [13]	Female sheep	3 years	8: 2 experimental groups of 4 sheep each	Load-bearing area of the medial femoral condyle	Full-thickness cartilage defect of 11 × 8 mm^2^	14 days (*n* = 2) and 3 months (*n* = 6)
Erggelet et al. [14]	Female sheep	3 years	8: 2 experimental groups of 4 sheep each	Load-bearing area of the medial femoral condyle	Full-thickness cartilage defect of 11 × 8 mm^2^	6 months
Getgood et al. [16]	Male goats	Skeletally mature, 50-79 kg	21: 12, collagen-GAG-CaP; 6, PLGA-CaS; 3, empty	(1) Medial femoral condyle (2) Lateral trochlear sulcus	Diameter: 5.8 mm Depth: 6 mm	12 and 26 weeks
Kon et al. [17]	Goats	Skeletally mature, 55 ± 11 kg	14 goats = 28 defects 7: drilled channels in the cartilage phase 8: drilled channels in the bone phase 8: unmodified coral cylinders 4: empty control	Medial and lateral femoral condyles	Diameter: 6 mm Depth: 8 mm	6 months
Filardo et al. [18]	Sheep	Adult, 65 ± 5 kg	6: 2 control, 4 experimental	Medial and lateral condyle	Diameter: 7 mm Depth: 5 mm	6 months
Mohan et al. [23]	Female sheep	>3.5 years, 82.4 kg	18: 6, implants; 3, implants+TGF-*β*; 3, implants+IGF-1; 6, microfracture	Medial and lateral femoral condyles	Diameter: 6 mm Depth: 6 mm	52 weeks
Guo et al. [91]	Sheep: male or female	12 months, 20.2 ± 5.3 kg	28: 16 scaffold +cells; 8 scaffold only; 4 control group	Weight-bearing area of the medial femoral condyle	Diameter: 8 mm Depth: 4 mm	12 and 24 weeks
Dorotka et al. [92]	Sheep: male or female	3-5 years, 78 kg	27: 3 experimental groups of 7 sheep each, 1 group 6 sheep	Medial femoral condyle	Diameter: 4.5 mm Depth: to the tidemark bone	4 and 12 months
Lind et al. [93]	Goats	Adult	20: 4 experimental groups of 5 goats each	Medial femoral condyle	Full-thickness cartilage defect Diameter: 6 mm	4 months
Lind and Larsen [94]	Goats	Adult	8: 2 experimental groups of 4 goats each	Weight-bearing area of the medial femoral condyle	Diameter: 5 mm Depth: to the subchondral bone	4 months
Gille et al. [95]	Female sheep	12 and 18 months, 46 ± 9.6 kg	30: 5 experimental groups of 6 sheep each	Medial femoral condyle and lateral distal facet of the trochlea	Diameter: 7 mm Calcified cartilage removed without damaging the subchondral bone	12 months
Hao et al. [96]	Sheep: male or female	12 months, 20-25 kg	24: 12 scaffold+cells, 2 control groups of 6 sheep each	Nonweight-bearing area of the medial femoral condyle	Diameter: 8 mm Depth: 3 mm	12 and then 24 weeks
Leng et al. [97]	Male goats	1.5-2 years, 18-25 kg	56: 4 experimental groups of 14 goats each	Weight-bearing region of the medial femoral condyle	Diameter: 6 mm Depth: 5 mm	16 weeks
Bernstein et al. [98]	Female sheep	2-4 years, 74.54 ± 16.55 kg	28: 4 time points with 7 sheep each	Load-bearing area of the medial femoral condyle	Diameter: 7 mm Depth: 25 mm	6, 12, 26, and 52 weeks
Schleicher et al. [99]	Female Merino sheep	3 years, 58.4 ± 9.3 kg	12: 6, 6 weeks; 3, 3 months; 3, 6 months	Weight-bearing area of the medial femoral condyle	Diameter: 9.5 mm Depth: 11 mm	6 weeks 3 months 6 months
Pei et al. [100]	Goats		12: 3 experimental groups of 4 goats each	Weight-bearing area of the medial femoral condyle	Diameter: 6 mm Depth: 12 mm	12 weeks 24 weeks
Hoppe et al. [101]	Sheep	3-5 years	24: 4 experimental groups of 6 sheep each	Medial femoral condyle	Diameter: 6 mm Depth: 8 mm	26 weeks
Zorzi et al. [63]	Female sheep	3-5 years, 58.02 ± 11.38 kg	15: 3 experimental groups of 5 sheep each	Medial femoral condyle	Diameter: 6 mm	6 months
Manunta et al. [102]	Female sheep	5.5 years, 45 kg	22: 1, 2, 6, and 12 months (4 for each) 24 months (6)	Medial femoral condyles	Diameter: 6 mm Depth: 2 mm	1, 2, 6, 12, and 24 months
Zhang et al. [103]	Goats	>12 months, 55 ± 11 kg	8 = 16 defects 2 groups 2 time points *n* = 4 each	Medial femoral condyle	Diameter: 6 mm Depth: 8 mm	12 and then 24 weeks
Di Bella et al. [104]	Male sheep	2-3 years, 69 kg	6: each sheep received four treatments	Weight-bearing surface of the lateral and medial condyles	Full-thickness chondral defect Diameter: 8 mm	8 weeks
Zhang et al. [105]	Male goats	Mature, 30 ± 5 kg	6: 2 experimental groups of 3 goats each	Lateral and medial femoral condyles	Diameter: 6.5 mm Depth: 1 mm	9 months
Bornes et al. [64]	Female sheep	2.6 ± 0.3 years, 75.0 ± 5.6 kg	5 sheep = 20 defects 2 experimental groups of 8 defects each 2 control groups of 2 defects each	Weight-bearing surface of the lateral and medial condyles	Diameter: 6. 95 mm	6 months
Jia et al. [106]	Goats	2-3 years, 58.4 ± 7.5 kg	24 goats = 48 defects 18: MLS 18: BLS 12: control	Medial femoral condyle	Diameter: 6 mm Depth: 8 mm	12, 24, and 48 weeks
Zhai et al. [75]	Goats	10 months, 20-25 kg	80: 4 experimental groups of 20 sheep each	Medial femoral condyle	Diameter: 6 mm Depth: 9 mm	6 months
Kon et al. [26]	Female sheep	Adult, 70 ± 5 kg	12: 3 experimental groups of 4 sheep each	Weight-bearing area of lateral and medial condyles	Diameter: 7 mm Depth: 9 mm	6 months
Getgood et al. [27]	Sheep	4.3 years	24: 4 experimental groups of 6 sheep each	(1) Medial femoral condyle (2) Lateral trochlear sulcus	Diameter: 5.8 mm Depth: 6 mm	26 weeks
Ivkovic et al. [107]	Female sheep	1-3 years	28: 10: TGF-*β*1 group 6: GFP group 6: BMC group 6: negative control group	Weight-bearing surface of the medial condyle	Partial-thickness chondral defect Diameter: 6.2 mm	6 months
Marmotti et al. [108]	Female goats	Adult	15: 3 experimental groups of 5 goats each	Central trochlear area	Diameter: 7 mm Depth: 3.5 mm	1, 3, 6, and 12 months
Schlichting et al. [60]	Male sheep	2-3 years, 77 ± 8 kg	24: 2 groups (3 and 6 months, *n* = 6 each)	Lateral and medial femoral condyles	Diameter: 7.3 mm Depth: 10 mm	3 and 6 months
Kon et al. [109]	Female goats	Mature female nonpregnant and nonlactating, 60 kg	8: with no control group	Medial femoral condyle	19 mm in length, 8 mm in width, 8 mm in height	12 months
Giretova et al. [110]	Sheep	2 years, 45-50 kg	6: with no control group	Medial femoral condyle	Diameter: 6 mm	6 months

**Table 2:** Elements to be considered in experimental design and the advantages of small ruminants in the corresponding element.

Elements of research design					Advantages of small ruminants
Research design	Scaffold	Material	Natural synthetic		Sufficient lifespan to observe degradation and toxicity
		Loaded ingredient	Chondrocytes MSCs PRP or others		Sufficient tissue and blood volumes for cell isolation
	Implementation	Group design	Animal number endpoints		(1) Cost effective (2) Less ethical issues (3) Flexible endpoint setting
		Defect creation	Location	Trochlea Condyle	Similar anatomy to human, thicker cartilage, dense bone layer, make defect creation more easily and precisely
			Range	Partial-thickness Full-thickness Osteochondral	
		Postoperative manage	Drugs	Pain controlling Antibiotics	Similar weight-bearing biomechanical environment to human, can imitate rehabilitation process Easy captivity
			Activity	Immobilization Restriction Move freely	
		Outcome assessment	Macroscopy Histology Imaging examination Biomechanics		Feasible to use routine diagnostic imaging and arthroscopy

## Supplementary Materials

Supplementary Materials A table shows information about tissue engineering cell-scaffold complexes used in small ruminant models.

## References

[1] Johnstone B., Alini M., Cucchiarini M. (2013). Tissue engineering for articular cartilage repair--the state of the art. *European Cells & Materials*.

[2] Anderson D. D., Chubinskaya S., Guilak F. (2011). Post-traumatic osteoarthritis: improved understanding and opportunities for early intervention. *Journal of Orthopaedic Research*.

[3] Music E., Futrega K., Doran M. R. (2018). Sheep as a model for evaluating mesenchymal stem/stromal cell (MSC)-based chondral defect repair. *Osteoarthritis and Cartilage*.

[4] Ahern B. J., Parvizi J., Boston R., Schaer T. P. (2009). Preclinical animal models in single site cartilage defect testing: a systematic review. *Osteoarthritis and Cartilage*.

[5] Quigley M. (2007). Non-human primates: the appropriate subjects of biomedical research?. *Journal of Medical Ethics*.

[6] Libbin R. M., Rivera M. E. (1989). Regeneration of growth plate cartilage induced in the neonatal rat hindlimb by reamputation. *Journal of Orthopaedic Research*.

[7] Chu C. R., Szczodry M., Bruno S. (2010). Animal models for cartilage regeneration and *repair*. *Tissue Engineering Part B: Reviews*.

[8] Allen M. J., Houlton J. E. F., Adams S. B., Rushton N. (1998). The surgical anatomy of the stifle joint in sheep. *Veterinary Surgery*.

[9] Proffen B. L., McElfresh M., Fleming B. C., Murray M. M. (2012). A comparative anatomical study of the human knee and six animal species. *The Knee*.

[10] de Barros C. N., Miluzzi Yamada A. L., Junior R. S. F. (2016). A new heterologous fibrin sealant as a scaffold to cartilage repair-experimental study and preliminary results. *Experimental Biology and Medicine (Maywood, N.J.)*.

[11] Beck A., Murphy D. J., Carey-Smith R., Wood D. J., Zheng M. H. (2016). Treatment of articular cartilage defects with microfracture and autologous matrix-induced chondrogenesis leads to extensive subchondral bone cyst formation in a sheep model. *The American Journal of Sports Medicine*.

[12] Seedhom B. B., Luo Z. J., Goldsmith A. J., Toyoda T., Lorrison J. C., Guardamagna L. (2007). In-situengineering of cartilage repair: a pre-clinicalin-vivoexploration of a novel system. *Proceedings of the Institution of Mechanical Engineers. Part H*.

[13] Erggelet C., Neumann K., Endres M., Haberstroh K., Sittinger M., Kaps C. (2007). Regeneration of ovine articular cartilage defects by cell-free polymer-based implants. *Biomaterials*.

[14] Erggelet C., Endres M., Neumann K. (2009). Formation of cartilage repair tissue in articular cartilage defects pretreated with microfracture and covered with cell-free polymer-based implants. *Journal of Orthopaedic Research*.

[15] Xia H., Li X., Gao W. (2018). Tissue repair and regeneration with endogenous stem cells. *Nature Reviews Materials*.

[16] Getgood A. M., Kew S. J., Brooks R. (2012). Evaluation of early-stage osteochondral defect repair using a biphasic scaffold based on a collagen-glycosaminoglycan biopolymer in a caprine model. *The Knee*.

[17] Kon E., Filardo G., Robinson D. (2014). Osteochondral regeneration using a novel aragonite-hyaluronate bi-phasic scaffold in a goat model. *Knee Surgery, Sports Traumatology, Arthroscopy*.

[18] Filardo G., Perdisa F., Gelinsky M. (2018). Novel alginate biphasic scaffold for osteochondral regeneration: an *in vivo* evaluation in rabbit and sheep models. *Journal of Materials Science. Materials in Medicine*.

[19] Cunniffe G. M., Díaz-Payno P. J., Sheehy E. J. (2019). Tissue-specific extracellular matrix scaffolds for the regeneration of spatially complex musculoskeletal tissues. *Biomaterials*.

[20] Vikingsson L., Sancho-Tello M., Ruiz-Saurí A. (2015). Implantation of a polycaprolactone scaffold with subchondral bone anchoring ameliorates nodules formation and other tissue alterations. *The International Journal of Artificial Organs*.

[21] Levingstone T. J., Ramesh A., Brady R. T. (2016). Cell-free multi-layered collagen-based scaffolds demonstrate layer specific regeneration of functional osteochondral tissue in caprine joints. *Biomaterials*.

[22] Getgood A., Henson F., Skelton C. (2014). Osteochondral tissue engineering using a biphasic collagen/GAG scaffold containing rhFGF18 or BMP-7 in an ovine model. *Journal of experimental orthopaedics*.

[23] Mohan N., Gupta V., Sridharan B. P. (2015). Microsphere-based gradient implants for osteochondral regeneration: a long-term study in sheep. *Regenerative Medicine*.

[24] Rhodes N. P., Srivastava J. K., Smith R. F., Longinotti C. (2004). Heterogeneity in proliferative potential of ovine mesenchymal stem cell colonies. *Journal of Materials Science. Materials in Medicine*.

[25] Oda K., Mori K., Imai S., Uenaka K., Matsusue Y. (2014). Comparison of repair between cartilage and osteocartilage defects in rabbits using similarly manipulated scaffold-free cartilage-like constructs. *Journal of Orthopaedic Science*.

[26] Kon E., Filardo G., Delcogliano M. (2010). Platelet autologous growth factors decrease the osteochondral regeneration capability of a collagen-hydroxyapatite scaffold in a sheep model. *BMC Musculoskeletal Disorders*.

[27] Getgood A., Henson F., Skelton C. (2012). The augmentation of a collagen/glycosaminoglycan biphasic osteochondral scaffold with platelet-rich plasma and concentrated bone marrow aspirate for osteochondral defect repair in Sheep. *Cartilage*.

[28] Jubel A., Andermahr J., Schiffer G. (2008). Transplantation of de novo scaffold-free cartilage implants into sheep knee chondral defects. *The American Journal of Sports Medicine*.

[29] Baghaban Eslaminejad M., Malakooty P. E. (2014). Mesenchymal stem cells as a potent cell source for articular cartilage regeneration. *World Journal of Stem Cells*.

[30] Uth K., Trifonov D. (2014). Stem cell application for osteoarthritis in the knee joint: a minireview. *World Journal of Stem Cells*.

[31] Pittenger M. F., Mackay A. M., Beck S. C. (1999). Multilineage potential of adult human mesenchymal stem cells. *Science*.

[32] Dominici M., le Blanc K., Mueller I. (2006). Minimal criteria for defining multipotent mesenchymal stromal cells. The International Society for Cellular Therapy position statement. *Cytotherapy*.

[33] Lee J. S., Im G. I. (2010). Influence of chondrocytes on the chondrogenic differentiation of adipose stem cells. *Tissue Engineering. Part A*.

[34] Reinwald S., Burr D. B., Duque G., Watanabe K. (2011). Other Large Animal Models. *Osteoporosis research--animal models*.

[35] Little C. B., Smith M. M., Cake M. A., Read R. A., Murphy M. J., Barry F. P. (2010). The OARSI histopathology initiative - recommendations for histological assessments of osteoarthritis in sheep and goats. *Osteoarthritis and Cartilage*.

[36] Dias I. R., Viegas C. A., Carvalho P. P. (2018). Large animal models for osteochondral regeneration. *Advances in Experimental Medicine and Biology*.

[37] Chevrier A., Kouao A. S. M., Picard G., Hurtig M. B., Buschmann M. D. (2015). Interspecies comparison of subchondral bone properties important for cartilage repair. *Journal of Orthopaedic Research*.

[38] Simon W. H. (1970). Scale effects in animal joints. I. Articular cartilage thickness and compressive stress. *Arthritis and Rheumatism*.

[39] Lu Y., Hayashi K., Hecht P. (2000). The effect of monopolar radiofrequency energy on partial-thickness defects of articular cartilage. *Arthroscopy*.

[40] Archibald M., Runciman J., Dickey J., Hurtig M. Do Animal Models Approximate the Sub-Chondral Bone and Cartilage Characteristics of Humans?.

[41] Brehm W., Aklin B., Yamashita T. (2006). Repair of superficial osteochondral defects with an autologous scaffold-free cartilage construct in a caprine model: implantation method and short-term results. *Osteoarthritis and Cartilage*.

[42] Reinholz G. G., Lu L., Saris D. B., Yaszemski M. J., O’Driscoll S. W. (2004). Animal models for cartilage reconstruction. *Biomaterials*.

[43] den Boer F. C., Patka P., Bakker F. C. (1999). New segmental long bone defect model in sheep: quantitative analysis of healing with dual energy X-ray absorptiometry. *Journal of Orthopaedic Research*.

[44] Johnson P. D., Besselsen D. G. (2002). Practical aspects of experimental design in animal research. *ILAR Journal*.

[45] Rudert M. (2002). Histological evaluation of osteochondral defects: consideration of animal models with emphasis on the rabbit, experimental setup, follow-up and applied methods. *Cells, Tissues, Organs*.

[46] Bate S., Karp N. A. (2014). A common control Group - Optimising the experiment design to maximise sensitivity. *PLoS One*.

[47] Hoemann C., Kandel R., Roberts S. (2011). International Cartilage Repair Society (ICRS) recommended guidelines for histological endpoints for cartilage repair studies in animal models and clinical trials. *Cartilage*.

[48] Lydon H., Getgood A., Henson F. M. D. (2019). Healing of osteochondral defects via endochondral ossification in an ovine model. *Cartilage*.

[49] Frisbie D. D., Oxford J. T., Southwood L. (2003). Early events in cartilage repair after subchondral bone microfracture. *Clinical Orthopaedics and Related Research*.

[50] Cook J. L., Hung C. T., Kuroki K. (2014). Animal models of cartilage repair. *Bone & Joint Research*.

[51] Hurtig M. B., Buschmann M. D., Fortier L. A. (2011). Preclinical studies for cartilage repair. *Cartilage*.

[52] Curl W. W., Krome J., Gordon E. S., Rushing J., Smith B. P., Poehling G. G. (1997). Cartilage injuries: A review of 31,516 knee arthroscopies. *Arthroscopy*.

[53] Hjelle K., Solheim E., Strand T., Muri R., Brittberg M. (2002). Articular cartilage defects in 1,000 knee arthroscopies. *Arthroscopy*.

[54] Madry H., Ochi M., Cucchiarini M., Pape D., Seil R. (2015). Large animal models in experimental knee sports surgery: focus on clinical translation. *Journal of Experimental Orthopaedics*.

[55] Frisbie D. D., Morisset S., Ho C. P., Rodkey W. G., Steadman J. R., Mcllwraith C. W. (2006). Effects of calcified cartilage on healing of chondral defects treated with microfracture in horses. *The American Journal of Sports Medicine*.

[56] Drobnič M., Radosavljevič D., Cör A., Brittberg M., Stražar K. (2010). Debridement of cartilage lesions before autologous chondrocyte implantation by open or transarthroscopic techniques. *Journal of Bone and Joint Surgery. British Volume (London)*.

[57] Miller D. J., Smith M. V., Matava M. J., Wright R. W., Brophy R. H. (2015). Microfracture and osteochondral autograft transplantation are cost-effective treatments for articular cartilage lesions of the distal femur. *The American Journal of Sports Medicine*.

[58] Orth P., Duffner J., Zurakowski D., Cucchiarini M., Madry H. (2016). Small-diameter awls improve articular cartilage repair after microfracture treatment in a translational animal model. *The American Journal of Sports Medicine*.

[59] Eldracher M., Orth P., Cucchiarini M., Pape D., Madry H. (2014). Small subchondral drill holes improve marrow stimulation of articular cartilage defects. *The American Journal of Sports Medicine*.

[60] Schlichting K., Schell H., Kleemann R. U. (2008). Influence of scaffold stiffness on subchondral bone and subsequent cartilage regeneration in an ovine model of osteochondral defect healing. *The American Journal of Sports Medicine*.

[61] Heckelsmiller D. J., James Rudert M., Baer T. E., Pedersen D. R., Fredericks D. C., Goetz J. E. (2017). Changes in joint contact mechanics in a large quadrupedal animal model after partial meniscectomy and a focal cartilage injury. *Journal of Biomechanical Engineering*.

[62] Erggelet C., Sittinger M., Lahm A. (2003). The arthroscopic implantation of autologous chondrocytes for the treatment of full-thickness cartilage defects of the knee joint. *Arthroscopy*.

[63] Zorzi A. R., Amstalden E., Plepis A. (2015). Effect of human adipose tissue mesenchymal stem cells on the regeneration of ovine articular cartilage. *International Journal of Molecular Sciences*.

[64] Bornes T. D., Adesida A. B., Jomha N. M. (2018). Articular cartilage repair with mesenchymal stem cells after chondrogenic priming: a pilot study. *Tissue Engineering. Part A*.

[65] Orth P., Goebel L., Wolfram U. (2012). Effect of subchondral drilling on the microarchitecture of subchondral Bone. *The American Journal of Sports Medicine*.

[66] Christ W., Lehnert T., Ulbrich B. (1988). Specific toxicologic aspects of the quinolones. *Reviews of Infectious Diseases*.

[67] Goebel L., Orth P., Müller A. (2012). Experimental scoring systems for macroscopic articular cartilage repair correlate with the MOCART score assessed by a high-field MRI at 9.4 T - comparative evaluation of five macroscopic scoring systems in a large animal cartilage defect model. *Osteoarthritis and Cartilage*.

[68] Fortier L. A., Mohammed H. O., Lust G., Nixon A. J. (2002). Insulin-like growth factor-I enhances cell-based repair of articular cartilage. *Journal of Bone and Joint Surgery. British Volume (London)*.

[69] Niederauer G. G., Slivka M. A., Leatherbury N. C. (2000). Evaluation of multiphase implants for repair of focal osteochondral defects in goats. *Biomaterials*.

[70] Brittberg M., Winalski C. S. (2003). Evaluation of cartilage injuries and repair. *The Journal of Bone and Joint Surgery. American Volume*.

[71] van den Borne M. P., Raijmakers N. J. H., Vanlauwe J. (2007). International Cartilage Repair Society (ICRS) and Oswestry macroscopic cartilage evaluation scores validated for use in autologous chondrocyte implantation (ACI) and microfracture. *Osteoarthritis and Cartilage*.

[72] Goldring S. R., Goldring M. B. (2016). Changes in the osteochondral unit during osteoarthritis: structure, function and cartilage-bone crosstalk. *Nature Reviews Rheumatology*.

[73] Wang F., Ying Z., Duan X. (2009). Histomorphometric analysis of adult articular calcified cartilage zone. *Journal of Structural Biology*.

[74] Roberts S., Menage J., Sandell L. J., Evans E. H., Richardson J. B. (2009). Immunohistochemical study of collagen types I and II and procollagen IIA in human cartilage repair tissue following autologous chondrocyte implantation. *The Knee*.

[75] Zhai C., Fei H., Hu J. (2018). Repair of articular osteochondral defects using an integrated and biomimetic trilayered scaffold. *Tissue Engineering. Part A*.

[76] O’Driscoll S. W., Keeley F. W., Salter R. B. (1988). Durability of regenerated articular cartilage produced by free autogenous periosteal grafts in major full-thickness defects in joint surfaces under the influence of continuous passive motion. A follow-up report at one year. *The Journal of Bone and Joint Surgery. American Volume*.

[77] Mainil-Varlet P., van Damme B., Nesic D., Knutsen G., Kandel R., Roberts S. (2010). A new histology scoring system for the assessment of the quality of human cartilage repair: ICRS II. *The American Journal of Sports Medicine*.

[78] Pineda S., Pollack A., Stevenson S., Goldberg V., Caplan A. (1992). A semiquantitative scale or histologic grading of articular cartilage repair. *Acta Anatomica (Basel)*.

[79] Rutgers M., van Pelt M. J. P., Dhert W. J. A., Creemers L. B., Saris D. B. F. (2010). Evaluation of histological scoring systems for tissue-engineered, repaired and osteoarthritic cartilage. *Osteoarthritis and Cartilage*.

[80] Novak T., Fites Gilliland K., Xu X. (2016). *In* VivoCellular infiltration and remodeling in a decellularized ovine osteochondral allograft. *Tissue Engineering. Part A*.

[81] Kurkijärvi J. E., Mattila L., Ojala R. O. (2007). Evaluation of cartilage repair in the distal femur after autologous chondrocyte transplantation using *T*_2_ relaxation time and dGEMRIC. *Osteoarthritis and Cartilage*.

[82] Poulet B., de Souza R., Kent A. V. (2015). Intermittent applied mechanical loading induces subchondral bone thickening that may be intensified locally by contiguous articular cartilage lesions. *Osteoarthritis and Cartilage*.

[83] Stok K. S., Lisignoli G., Cristino S., Facchini A., Müller R. (2009). Mechano-functional assessment of human mesenchymal stem cells grown in three-dimensional hyaluronan-based scaffolds for cartilage tissue engineering. *Journal of Biomedical Materials Research. Part A*.

[84] Pobloth A. M., Johnson K. A., Schell H. (2016). Establishment of a preclinical ovine screening model for the investigation of bone tissue engineering strategies in cancellous and cortical bone defects. *BMC Musculoskeletal Disorders*.

[85] Ude C. C., Shamsul B. S., Ng M. H. (2018). Long-term evaluation of osteoarthritis sheep knee, treated with TGF-*β*3 and BMP-6 induced multipotent stem cells. *Experimental Gerontology*.

[86] Lo Monaco M., Merckx G., Ratajczak J. (2018). Stem cells for cartilage repair: preclinical studies and insights in translational animal models and outcome measures. *Stem Cells International*.

[87] Moran C. J., Ramesh A., Brama P. A., O’Byrne J. M., O’Brien F. J., Levingstone T. J. (2016). The benefits and limitations of animal models for translational research in cartilage repair. *Journal of Experimental Orthopaedics*.

[88] Newman E., Turner A. S., Wark J. D. (1995). The potential of sheep for the study of osteopenia: current status and comparison with other animal models. *Bone*.

[89] Turner A. S. (2007). Experiences with sheep as an animal model for shoulder surgery: strengths and shortcomings. *Journal of Shoulder and Elbow Surgery*.

[90] Jackson D. W., Lalor P. A., Aberman H. M., Simon T. M. (2001). Spontaneous repair of full-thickness defects of articular cartilage in a goat Model. *The Journal of Bone and Joint Surgery. American Volume*.

[91] Guo X., Wang C., Duan C. (2004). Repair of osteochondral defects with autologous chondrocytes seeded onto bioceramic scaffold in sheep. *Tissue Engineering*.

[92] Dorotka R., Bindreiter U., Macfelda K., Windberger U., Nehrer S. (2005). Marrow stimulation and chondrocyte transplantation using a collagen matrix for cartilage repair. *Osteoarthritis and Cartilage*.

[93] Lind M., Larsen A., Clausen C., Osther K., Everland H. (2008). Cartilage repair with chondrocytes in fibrin hydrogel and MPEG polylactide scaffold: an *in vivo* study in goats. *Knee Surgery, Sports Traumatology, Arthroscopy*.

[94] Lind M., Larsen A. (2008). Equal cartilage repair response between autologous chondrocytes in a collagen scaffold and minced cartilage under a collagen scaffold: an *in vivo* study in goats. *Connective Tissue Research*.

[95] Gille J., Kunow J., Boisch L. (2010). Cell-laden and cell-free matrix-induced chondrogenesis versus microfracture for the treatment of articular cartilage Defects. *Cartilage*.

[96] Hao T., Wen N., Cao J. K. (2010). The support of matrix accumulation and the promotion of sheep articular cartilage defects repair *in vivo* by chitosan hydrogels. *Osteoarthritis and Cartilage*.

[97] Leng P., Ding C. R., Zhang H. N., Wang Y. Z. (2012). Reconstruct large osteochondral defects of the knee with hIGF-1 gene enhanced Mosaicplasty. *The Knee*.

[98] Bernstein A., Niemeyer P., Salzmann G. (2013). Microporous calcium phosphate ceramics as tissue engineering scaffolds for the repair of osteochondral defects: histological results. *Acta Biomaterialia*.

[99] Schleicher I., Lips K. S., Sommer U. (2013). Allogenous bone with collagen for repair of deep osteochondral defects. *The Journal of Surgical Research*.

[100] Pei Y., Fan J. J., Zhang X. Q., Zhang Z. Y., Yu M. (2014). Repairing the osteochondral defect in goat with the tissue-engineered osteochondral graft preconstructed in a double-chamber stirring bioreactor. *BioMed Research International*.

[101] Hopper N., Wardale J., Brooks R., Power J., Rushton N., Henson F. (2015). Peripheral blood mononuclear cells enhance cartilage repair in *in vivo* osteochondral defect model. *PLoS One*.

[102] Manunta A. F., Zedde P., Pilicchi S. (2016). The use of embryonic cells in the treatment of osteochondral defects of the knee: an ovine *in vivo* study. *Joints*.

[103] Zhang T., Zhang H., Zhang L. (2017). Biomimetic design and fabrication of multilayered osteochondral scaffolds by low-temperature deposition manufacturing and thermal-induced phase-separation techniques. *Biofabrication*.

[104] di Bella C., Duchi S., O'Connell C. D. (2018). In situhandheld three-dimensional bioprinting for cartilage regeneration. *Journal of Tissue Engineering and Regenerative Medicine*.

[105] Zhang Y., Liu S., Guo W. (2018). Human umbilical cord Wharton's jelly mesenchymal stem cells combined with an acellular cartilage extracellular matrix scaffold improve cartilage repair compared with microfracture in a caprine model. *Osteoarthritis and Cartilage*.

[106] Jia S., Wang J., Zhang T. (2018). Multilayered scaffold with a compact interfacial layer enhances osteochondral defect repair. *ACS Applied Materials & Interfaces*.

[107] Ivkovic A., Pascher A., Hudetz D. (2010). Articular cartilage repair by genetically modified bone marrow aspirate in sheep. *Gene Therapy*.

[108] Marmotti A., Bruzzone M., Bonasia D. E. (2013). Autologous cartilage fragments in a composite scaffold for one stage osteochondral repair in a goat model. *European Cells & Materials Journal*.

[109] Kon E., Robinson D., Shani J. (2020). Reconstruction of Large Osteochondral Defects Using a Hemicondylar Aragonite- Based Implant in a Caprine Model. *Arthroscopy*.

[110] Giretova M., Medvecky L., Petrovova E. (2019). Polyhydroxybutyrate/chitosan 3D scaffolds promote *in vitro* and *in vivo* chondrogenesis. *Applied Biochemistry and Biotechnology*.

