# Supplementary material for: Small Ruminant Models for Articular Cartilage Regeneration by Scaffold-Based Tissue Engineering
Source: Stem Cells Int. 2021 Dec 6;2021:5590479. doi: 10.1155/2021/5590479 (PMC8668357; doi:10.1155/2021/5590479)
Supplement: Supplementary Materials — A table shows information about tissue engineering cell-scaffold complexes used in small ruminant models. [file 5590479.f1.doc]

Cell type, scaffold material, cell concentration and mode of loading.

| **Reference** | **Scaffold material** | **Cell** | **Seeding density** | **Cell-scaffold construction** |
| --- | --- | --- | --- | --- |
| Niederauer et al. | 1: 75PLA: 25 PGA  2: 20% PGA fibers - 75:25 PLG  3: 55:45 PLG+20% Bioglass  4: 75:25 PLG+50%MGCS (medical-grade calcium sulfate) | Autologous costochondral chondrocytes | 30, 490±11, 409 | Co-culture  48 h |
| Guo et al. | Porous β-TCP bioceramic | Autologous bone marrow-derived mesenchymal stem cells (BMSCs)  Passage 3 | 3×10^7^ | Inoculated into scaffold  Co-culture  24 h |
| Guo et al. | Porous β-TCP bioceramic | Autologous chondrocytes from the humeral head  Passage 3 | 5×10^7^ | Co-culture  24 h |
| Dorotka et al. | Porcine collagen matrix  Type I/III collagen  layer  Type II collagen layer | Autologous chondrocytes  Passage 2-3 | 1×10^6^ | Dropwise fashion  Co-culture  48 h |
| Lind et al. | MPEG-PLGA | Autologous chondrocytes  Passage 1-2 | 1×10^6^ | Resuspended in fibrinogen solution before seeded onto the MPEG-PLGA scaffolds  4 weeks |
| Kon et al. | Nucleating collagen fibrils with HA nanoparticles | Autologous chondrocytes | 0.5-1.0×10^7^ | Dropwise onto matrix surface  Co-culture  5 days |
| Lind and Larsen. | Porcine type I and III collagen (Chondro-Gide, Geisthlich) | Autologous  chondrocytes  Passage 1-2 | 1×10^6^ | 3-4 weeks  Injected onto scaffold  5 min before it was placed |
| Gille et al. | Collagen I/III, Chondro-Gide®; collagen II, Chondrocell® | Autologous chondrocytes | 1×10^5^ | Seeded on collagen scaffolds  3 additional days |
| Hao et al. | Chitosan hydrogel | Autologous chondrocytes  Passage 3 | 4×10^7^ | Mixed with 1 ml chitosan hydrogel  1 day |
| Leng et al. | Calcium alginate gel | BMSCs with and without modified hIGF-1 gene | 3×10^7^ | Mixture |
| Bernstein et al. | β-TCP ceramic | Autologous chondrocytes | 1×10^5^ | Co-culture  4 weeks |
| Schleiche et al. | Allogenous bone cylinders  Upper layer of porcine collagen | Autologous chondrocytes |  | Dripped onto scaffold with implantation in the scaffold |
| Pei et al. | β-TCP | Autologous BMSCs  Passage 3 |  | Culture in bioreactor  2 weeks |
| Caminal et al. | PLGA | Autologous mesenchymal stromal cells | 3.3±0.4×10^6^ | Overnight  Co-culture |
| Caminal et al. | PLGA | Different cell sources [cartilage, fat and bone marrow] | Adipose tissue-derived stem cells (ASCs)：  1.0/1.1/2.6/3.5/4.5×10^6^  BMSCs：  1.4/3.2/5.0/4.9×10^6^  Chondrocytes  0.7/1.0/1.1/1.3/2.4/3.7/3.9/4.0/4.2×10^6^ | Co-culture  24 h |
| Hopper et al. | Collagen-glycosaminoglycan (GAG) scaffold (ChondroMimetic, TiGenix) | Autologous peripheral blood mononuclear cells (PBMCs)  Ovine BMSCs | BMSCs: 1×10^6^  PBMCs: 2×10^5^ | BMSCs: Co-culture, 24 h  PBMCs: Added to the scaffold during the surgery |
| Zorzi et al. | Chitosan and type I collagen | Human adipose tissue mesenchymal stem cells  Passage 4 | 1×10^6^ | Co-culture  3 days |
| Manunta et al. | Fibrin glue | Embryonic stem-like cells | 5-7×10^5^ | Embedded in fiber glue |
| Zhang et al. | Cartilage ECM-derived layer: cartilage matrix and chitosan  Compact layer: PLGA/TCP  Bone layer: 3D-printed PLGA/TCP+type I collagen | Autologous  BMSCs  Passage 3 | 1×10^6^ | Co-culture  3 days |
| Di Bella et al. | Biopen  Gelatin methacrylamide (GelMa) and HYA methacrylate hydrogel | Allogeneic adipose-derived MSCs  Passage 3 | 2.5x10^6^/mL | Mixture |
| Zhang et al. | Acellular cartilage ECM-oriented scaffold | Human umbilical cord Wharton's jelly MSCs  Passage 3 | 1×10^6^ | Co-culture  3 days |
| Bornes et al. | Hyaluronic acid scaffold | Autologous  BMSCs  Passage 2 | 1×10^7^ | Co-culture  4 days under normoxia (21% oxygen) or hypoxia (3% oxygen) |
| Jia et al. | Oriented articular cartilage ECM (bovine)-derived  cartilage layer, an intermediate compact interfacial layer, and a 3DP porous PLGA/TCP bone layer | BMSCs | 5×10^5^ | Implanted on each cartilage and bone scaffold |
| Zhai et al. | Cartilage layer: 0.5% (w/v) chitosan/gelatin solution Dense isolation layer: 2% (w/v) chitosan/gelatin solution Subchondral bone layer: β-TCP: | Autologous BMSCs  Autologous chondrocytes  Passage 2 | 1.5×10^4^ | Co-culture  5 weeks  MSCs were seeded in the subchondral bone layer before graft implantation |
| Vahedi et al. | PCL | ASCs | 1×10^6^ | Co-culture  1 week |
| Wei et al. | Cartilage layer: collagen membranes  Subchondral bone layer: porous tantalum | Cartilage layer:: chondrocytes  Subchondral bone layer: autologous BMSCs | Chondrocytes：  8×10^7^ cells/mL  BMSCs：  6×10^7^ cells/mL | Co-culture  21 days |
